# Can Liquid Lenses Increase Depth of Field in Head Mounted Video See-Through Devices?

**DOI:** 10.3390/jimaging7080138

**Published:** 2021-08-05

**Authors:** Marina Carbone, Davide Domeneghetti, Fabrizio Cutolo, Renzo D’Amato, Emanuele Cigna, Paolo Domenico Parchi, Marco Gesi, Luca Morelli, Mauro Ferrari, Vincenzo Ferrari

**Affiliations:** 1 Department of Information Engineering, University of Pisa, 56122 Pisa, Italy; d.domeneghetti92@gmail.com (D.D.); fabrizio.cutolo@endocas.unipi.it (F.C.); renzo.damato@endocas.unipi.it (R.D.); vincenzo.ferrari@unipi.it (V.F.); 2 EndoCAS Center, Department of Translational Research and New Technologies in Medicine and Surgery, University of Pisa, 56122 Pisa, Italy; mauro.ferrari@unipi.it; 3 Department of Translational Research and New Technologies in Medicine and Surgery, University of Pisa, 56122 Pisa, Italy; emanuele.cigna@unipi.it (E.C.); paolo.parchi@unipi.it (P.D.P.); marco.gesi@med.unipi.it (M.G.); luca.morelli@unipi.it (L.M.)

**Keywords:** head mounted display, magnification, autofocus, Video See-Through, liquid lenses

## Abstract

Wearable Video See-Through (VST) devices for Augmented Reality (AR) and for obtaining a Magnified View are taking hold in the medical and surgical fields. However, these devices are not yet usable in daily clinical practice, due to focusing problems and a limited depth of field. This study investigates the use of liquid-lens optics to create an autofocus system for wearable VST visors. The autofocus system is based on a Time of Flight (TOF) distance sensor and an active autofocus control system. The integrated autofocus system in the wearable VST viewers showed good potential in terms of providing rapid focus at various distances and a magnified view.

## 1. Introduction

The use of magnifying glasses in surgical practice has been well established [1]. The magnification depends on the surgical specialties, the intervention and also the device deployed to change the magnified view. For high magnifications, 10× microscopes are used and the surgeon is in a sitting position with his/her arms resting on a support, in order to prevent tremors and maximize the accuracy of the intervention [2]. For surgical practices that require limited magnifications, the ideal position of the surgeon is upright and a wearable device is the best solution. The surgical loupes currently on the market provide magnifications from 1.5× to 5× and several working distances. The working distance depends on the focal length of the device employed, and the optical properties of the device also define the depth of field (DOF), i.e., the distance between the nearest and farthest plane in focus in an image (e.g., from 30 cm to 45 cm). This intrinsically depends on the choice of optics and camera sensor and defines the distance limits at which a camera is able to keep objects in focus [3,4]. The DOF for near working distances is limited, about 15 cm for lower magnifications, and decreases as the magnification increases [5,6]. Ideally in a surgical scenario, the surgeon should wear a magnifying device capable of focusing on things close by (e.g., the surgeon’s hands) as well as those further away (e.g., a nurse handing him/her a surgical instrument). This is not possible with modern surgical loupes.

Wearable magnifying devices with a wider depth of field would increase the comfort and number of users of magnifying devices. The solution proposed in this paper is to integrate an automatic focusing system within a head mounted display Video See-Through device, so that the DOF imposed by the choice of hardware (optics and sensor) can move back and forth in order to focus on a selected area to be magnified.

Head Mounted Displays (HMDs) are wearable devices that provide the users with an enhanced perception of the real world thanks to semi-transparent displays put in front of their eyes. There are two categories depending on the visualization paradigm. Video See-Through (VST) systems stream on micro displays inside the HMD the video feeds from cameras mounted on the HMD, and the rendering of virtual objects is combined with the scene captured by the cameras via software. Optical See-Through (OST) systems have a light beam combiner, which guides the virtual content to the user’s eye, e.g., a semi-transparent mirror, which reflects light from the micro-display with the virtual information, and transmits light from the real world [7].

There are several solutions to the problem of limited DOF. One exciting technological solution has been the Varioscope^®^, an HMD OST used in surgery [8,9] which provides variable zoom and focus, but is limited by a speed of focus that is not transparent to the user. Martin-Gonzalez et al. in [10] propose a HMD VST device that offers a digital zoom dependent on the framed area.

We used an HMD VST device to provide magnification because, as previously mentioned, the view provided by these devices is dependent on the camera and display features. Consequently, the vision is directly conditioned by the characteristics of the (i) HMD displays (resolution and image quality), (ii) cameras (resolution, frames per second, ability to focus) and (iii) lenses (depth of field, geometric and chromatic aberrations introduced by the optics of the camera). This enables the device’s features to be customized and to easily integrate a new focusing system, based on an active focus system and liquid lens optics, aimed at switching the focus at a speed that is transparent to the human eye.

### Background and Related Work

The human eye can change the focusing distance through an autonomous mechanism called accommodation. During accommodation, the eye varies the anterior curvature of the lens through the ciliary muscle, in order to correctly focus the images on the retina. Accommodation is hard to measure because there are other physiological phenomena that occur concurrently, eye vergence [11], myosis and mydriasis, which can affect the DOF [3]. Age and lighting condition also affect the accommodation [12]. In [13,14], accommodation is insulated by working with one eye and with pupil dilation. The results show that the accommodation time is composed of reaction time and response time. Both articles agree that reaction times are in the range of about 200 to 400 ms. In [14], the response time is about 400 to 1000 ms and in [13], the reaction time is about 300 ms and the response time is comparable with the time presented in [14]. In general, as focusing is dependent on different factors, a focusing time of <200 will be transparent to the user.

The objective of an autofocus system, integrated into a VST visor, is to reproduce the capacity of the human eye in terms of focusing speed.

According to the control method used, autofocus systems are divided into passive and active. Passive control systems are based on the continuous control of image sharpness. Active control systems are based on direct or indirect distance measurements obtained with a distance sensor: ultrasonic, infrared or Time of Flight [15]. Passive autofocus algorithms are divided into contrast/sharpness detection and phase detection: the former measures the sharpness of each frame for the best focus position, the latter measures the phase difference between two captured images in order to estimate the focus position. Contrast detection algorithms are divided into global search and local search and according to the function to maximize such as first-order derivatives, the absolute gradient, the Prewitt operator, the Robert operator and the Sobel operator, second-order derivatives, such as the Laplacian operator, and histogram-based, image-statistics-based, correlation-based and data-compression-based [16]. Both phase detection and contrast detection work as follows: (1) capture the image; (2) calculate a function that expresses the focus level; (3) move the lens and repeat.

On the other hand, once the distance between the camera and the object is known, an active autofocus system moves the lens into the correct position.

To evaluate an autofocus system, the accurancy and speed need to be taken into account. Both are influenced by the algorithm used to find the focus position, the engine speed for moving the lens, the hardware computing power, image acquisition speed and lens characteristics (focal length, aperture).

In terms of the speed of the focusing systems the speed depends on several factors. For example, if the processor, the lens movement motor or the frame rate of the camera sensor is slow, the whole system will be slow. In addition, the literature reports the speeds in the various steps of the search algorithm, in order to compare different algorithms in passive autofocus systems. We report some numbers found in the literature to describe the time required for focusing on passive systems: at least 384.2 ms on average according to [17], which is similar to the results presented in [18]. In [16], the authors present a passive AF method that takes only three steps to find the optimum focus, unlike the algorithms in [19,20] that take about seven steps. The phase-detection algorithm is also very fast in terms of steps [21]. The time required for active systems is hard to determine as it depends on the lens actuator speed (voice coil motor, stepper motor, piezoelectric motor, dc motor…) and time needed to perform a distance measure. An active autofocus system was implemented in an LG smartphone which apparently can focus in 276 ms [22].

The focusing system proposed in this work is based on the use of liquid lenses [23].

A liquid lens consists of a circular sealed cell with a hydrophobic inner surface, containing two immiscible liquids of the same density: a conductive liquid (e.g., water solution) and an insulating liquid (e.g., oil). The two solutions must have the same density so that the two phases do not move while keeping the optical axis fixed, which enables the lens to be used regardless of the orientation held. The aqueous phase is immersed in the oil phase and, under conditions of mechanical equilibrium, the interface between the two liquids has a spherical shape and forms a natural diopter. The lens refracts the light according to the difference between the refractive indexes between the two liquids and the curvature of the interface surface between the two liquids contained in the lens. The variation in the focal length is controlled by electrowetting, which after supplying a voltage to the lens causes the wettability of the two liquids to change the contact surface between the two liquids, thus having a spherical shape with a variable radius of curvature. These lenses keep the optical axis stable thanks to centering by the geometry and centering by the distribution of the electric thickness gradient and without presenting any significant hysteresis [23,24,25].

Liquid lenses are suitable for integration into modern digital cameras with CMOS or CCD sensors to obtain autofocus systems [26]. Indeed, cameras with liquid lenses are already on the market [27,28,29]. After an extensive search on the market and in the literature, the following liquid lenses were selected: Corning^®^ Varioptic^®^ C-Module A-39N with a MAX14574 driver (Maxim Integrated, San Jose, CA, USA).

## 2. Materials and Methods

We developed a wearable system on the top of the OST HMD from the ARS 30 Trivisio^®^ model (www.trivisio.com, accessed on 21 April 2020). The OST visor was transformed into VST by mounting an obscuring medium (an electrically driven Optical Shutter by LCTech) and a couple of Leopard Imaging OV580 stereo cameras (Leopard Imaging Inc., Fremont, CA, USA) with liquid lens optics type A-39N with a focal length of 15.8 cm. The visor obtained provides stereoscopic view, 60 fps and 1280 × 1024 p resolution.

**Figure 1:**
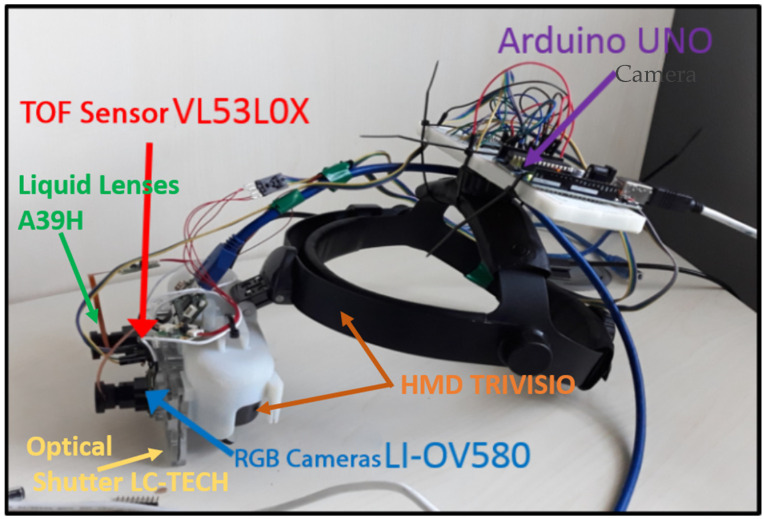
The Trivisio visor with the proposed autofocus system integrated.

**Figure 2:**
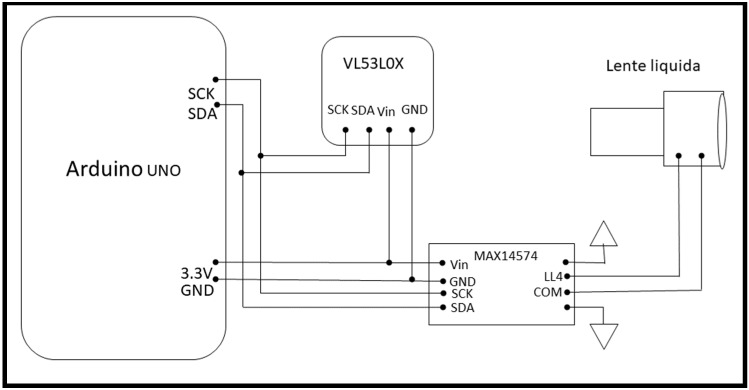
Autofocus system block diagram.

The autofocus system was mounted on the top of the visor, as shown in Figure 1. The system is composed of: VL53L0X Time Of Flight (TOF) sensor (Adafruit Industries, New York City, NY, USA), Arduino UNO control board (www.arduino.com, accessed on 11 May 2021), liquid lens optics and drivers from Maxim Integrated™, model MAX14574 (Maxim Integrated, San Jose, CA, USA), and the integrated driver converts digital values passed from the control board into analog voltages sent to the liquid lens. The autofocus system works as follows: the distance sensor, placed in front of the viewer, measures the distance from the framed object and sends it to the control board which, based on a look up table, decides the voltage to be sent to the lens. Figure 2 shows the connections between all the electronic components.

### 2.1. Visor Magnification

For VST visors, magnification is given by the ratio of the visor’s angle of view to the camera’s angle of view and the displacement between the user’s eye position and the camera’s position. Although the purpose of this work is not directly related to magnification, the idea is that the autofocus system developed could be integrated within a VST HMD that provides the surgeon with a zoom, and thus, we evaluated which magnification can be obtained with our system.

The camera, which enables the scene to be visualized, is in front of the eyes, which means that there is further magnification because it is as if the user was looking closer. The angle of view of the visor is the angle that the eye engages to look at the display. If the angle of view of the camera is less than the angle of view of the visor a smaller portion of space is seen, which will be reported on the same portion of the display with the same number of pixels and will appear enlarged. The field of view (FOV), expressed as the angle in degrees, of the camera is calculated considering the pinhole model of the camera with the following relationship:

(1) FOV=2 tan−1(d2f)

where *d* are the sensor dimensions (width, height, diagonal) and *f* is the focal length of the camera lens.

The magnification is given by the ratio between the size of the image that the user would see on the visor display, *i*, and the size of the image seen from the camera, *i′*, and is calculated with the following formula:

(2) M=ii′=x tan(FOVH2)(x−a)tan(FOVH′2)

where *a* is the forward displacement between the user’s eye and the camera’s center of projection, *FOV_H_* and *FOV′_H_* are the horizontal FOV of the camera and the horizontal FOV of the visor, respsectively.

Below are the results of the calculations of the theoretical magnification of our device starting with the following data: 5.12 mm active sensor width, 2.88 mm active sensor height, 9 cm distance between eye and the camera, 23.6° horizontal angle of view of the display and 15.8 mm focal length. Using (1), the angle of view of the camera can be set to 18.4°, then using (2), the total magnification of the visor is obtained, in this case, 1.67×. The magnification can be increased either using a digital zoom, losing image resolution or adapting the optic; however, as in this work our purpose was to widen the DOF, the result obtained is any case sufficient.

### 2.2. Liquid Lens Lookup Table Calibration

The processor decides which value to write in the driver’s register based on the distance measured by the sensor. The look up table (LUT) unambiguously matches a distance value to a digital value. An LUT between the distance from the camera and the voltage to be supplied to the lens was calibrated. The calibration was performed as follows: a calibration object (a chessboard with a square side of 1 cm) was placed at various distances, from 10 cm to 120 cm, at regular intervals of 5 cm. For each distance, the voltage applied to the lens was varied until the image was correctly focused (the correct focus was visually controlled by three operators). A different LUT was calibrated for each camera (right and left eye). In order to make the transition from one digital level to another as smooth as possible and to improve the user experience, we have interpolated the LUT values with a linear function in stretches. In particular, the interpolation is linear within the intervals. At each specific distance, one digital value corresponds between the extreme digital values of the range.

One important thing to note is that liquid lenses change dioptric power according to their temperature. However, during the tests performed, it was not necessary to recalibrate the lookup table as the control electronics were positioned away from the lens which remained at room temperature. Working thus at constant temperatures, there were no changes in the dioptric power of the lens.

### 2.3. Testing Set Up

The system was tested to assess the autofocus system performance in terms of focus speed. It was also tested by 20 clinicians to qualitatively assess its usability and comfort, including five surgeons with different surgical specialties.

#### 2.3.1. Quantitative Test

The following test was performed to measure the focusing speed of the autofocus system integrated into the visor. The visor was placed and fixed on a support at a 30 cm distance from an object with a clear geometrical pattern (thus, easy to understand if it is in focus), and a second object was placed behind the first object, 60 cm away from the visor. The test consisted in the almost instantaneous removal of the nearest object from the scene and its relocation. The operation was repeated several times and the point of view of the visor was recorded by the camera with a frame rate of 90 frames per second, while the distance sensor performed the measures every 50 ms (The VL53L0X allows us to select the time needed for a distance measurement; we have chosen to set it to 50 ms because it is a fair compromise between measurement speed and measurement accuracy). The test lasted two minutes. The focus time was measured by counting the frames needed to switch between the first and the second object in focus and vice versa. When switching focus from near to far, the frames counted are those included from the moment the closest object is removed (completely disappeared from the camera image) to when the second object appears completely in focus. When switching focus from far to near, frames are considered from the time when the near object completely covers the scene to when it first appears in focus.

Theoretically, the focus time indicated with *Tf* is composed of: the time taken by the distance sensor to perform a reading indicated with *T*r, the *Tes* code execution time, the time the driver takes to supply the voltage to the lens Ts, and the time taken by the lens to deform and change its focal length *Tlens*. The *Tlens* depends on how big the dioptric step is, expressed in Diopter D. In this test, the lens covers a 3D interval to perform the focusing. *Tf* is expressed by the following relationship:

*Tf* = *T*r + *Tes* + Ts + *Tlens* (3)

Control board execution time, *Tes*, is 7 ms (we calculated it with millis () command in Arduino IDE). Ts is very small and therefore negligible.

#### 2.3.2. Qualitative Usability Tests

**Figure 3:**
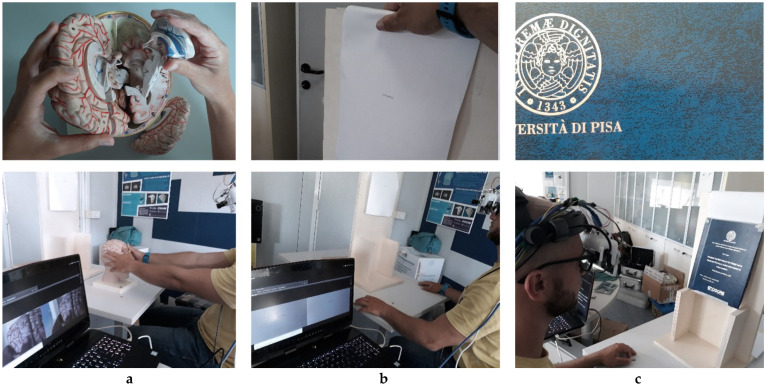
(**a**) First task—a user reassembles an anatomical brain phantom, shown in the image above. (**b**) Second task—a user tries to read a sequence of characters, in the image above, and for each distance tested, the characters sizing varied in order to always be the same size as the eye of the user. (**c**) Third task—the texturized object on the right of the image is moved backward and forward while the user tries to keep focusing on all the details of the leather cover.

Three tests were carried out on the ability to focus, the perceived focus speed, the image quality and the ability to provide a stereoscopic view of the display over a wide range of distances (30–90 cm peri-personal area). The first test consisted of a manual task. Once the user was wearing the visor correctly, they were asked to take a phantom model of an assembled anatomical part (brain), separate each component, reassemble it and reinsert it in its place (Figure 3a). In this test, the user was free to look wherever they wanted and the time of execution of the task was not evaluated. The second test consisted of a reading task: seven sequences of five alphanumeric characters Figure 3b were presented to the user, who had to read the sequences. The sequences were presented, using a mobile support, by varying the distance from 30 to 90 cm with 10 cm intervals. The character sequences were different for each distance and had an increasing size proportionally to the distance so that, to be read, a visual acuity of at least 7/10 was sufficient according to the Snellen scale. The selected range of distances is not covered by a single DOF, and for each reading, the system updates the lens curvature in order to guarantee that the image is correctly focused. With a fixed focus, it would be impossible to read all the sequences in focus at all the proposed distances. The third task was a dynamic task: the user was shown an object with a high degree of detail. The object was placed on a mobile support and was moved in the range of 30–90 cm in front of the user while they tried to keep all the details in focus. The aim of the third test was to assess wheter the focusing speed of the system was sufficiently transparent to the user, Figure 3c. These three tasks were carried out by two non-homogeneous groups: group one—non surgeons (engineers and clinicians), group two—surgeons.

The first and third tests were evaluated by the user via a qualitative questionnaire based on a monotone Likert scale with five intervals [30]. The second test was evaluated by counting the reading errors made. The questionnaires assessed the visor’s ability to focus, the speed of focus, the resolution of the device, the ability to offer a stereoscopic view. For each statement in the questionnaire, the user could express his/her degree of agreement/disagreement on a five-step scale with a score from 1 (totally disagree) to 5 (totally agree). The reading task aimed to highlight the ability of the device to focus on different distances outside a single depth of field. Without a change in focal distance, it would be impossible to read the sequences of characters at each of the distances set in the task. This task also provides more precise information on the device’s angular resolution, because its visual acuity is not the same as the human eye.

The statements made in the questionnaires are reported in the tables in Section 3.

## 3. Results

### 3.1. Quantitative Test Results

To extract the results, two authors independently analyzed the obtained video in two separate sessions, so that they were not affected by each other’s judgments. A focusing event occurred approximately every two seconds. Both authors found 37 near to far focus events (N2F event) and 36 far to near focus events (F2N event). Data analysis was performed with IBM SPSS Statistics. The Shapiro Wilk’s normality test showed that the times and number of frames required for focusing had a non-Gaussian distribution for both N2F and F2N events. Mann-Whitney’s test for independent samples shows that there is no statistical difference in the times and number of frames required for focusing for both N2F and F2N events. The Wilcoxon test showed no significant differences in the time/frame required for focusing measured by each author. Table 1 reports the test results.

The results show that the autofocus system can focus in a working distance range between 30 cm and 60 cm, in 130 ms (average time).

### 3.2. Qualitative Test Results

The answers to the questionnaires were analyzed using IBM SPSS Statistics. In addition to the statements, the questionnaire collected personal data: age, gender, visual acuity (10/10 or less), occupation and level of experience with HMD-VST devices. The questionnaire was completed by 20 people aged between 25 and 67 with an average age of 38. This group included 5 surgeons and 15 non-surgeons: researchers, clinicians or biomedical engineering students.

Table 2 shows the results of the questionnaire for each statement. Since the results of the statements had no normal distribution, they are reported as the median and interquartile range for each statement; the distribution of the responses was verified as not normal, using the Shapiro-Wilk normality test. By performing the Wilcoxon test for all statements in the first task, we verified that statements (1), (2), (5), (7) are statistically significant and they express a positive judgment for the above statements. For statement (3), the test participants’ opinions were divided equally between positive and negative. This is probably due to their attitude towards the perception of the stereoscopic image. For statement (4), most of the participants remained neutral, and the others expressed both positive and negative opinions. For statements (6), as expected, the number of responses was insufficient to make a judgement (only surgeons answered this statements); however, in any case, the tendency showed positive results. The results of the Wilcoxon test for the third task showed that statements (2), (4)–(7) were statistically significant. For statements (1) and (3), due to the scarcity of participating subjects, the Wilcoxon test showed no statistical significance. However, there was a slight majority of positive opinions.

The Mann-Whitney test showed no statistical difference between the answers given by the sub-sample of non-surgeons and surgeons (as shown in Table 3).

This reinforces the overall results, as it underlines that educational/professional background is not significant to understand the test conducted and the assessments made. We also ran the Mann-Whitney test to verify whether there was a statistical difference between the answers given by the sub-sample who said they had good experience with VST devices and augmented reality and those who said they had no experience.

No statistical differences were found between the two groups for both tasks (as shown in Table 4). Again, previous expertise in wearable visors and augmented reality did not affect the test results, thus underlining the absolute value of the results.

For the reading task, we counted the number of reading errors and missed readings. With the Shapiro-Wilk test, we verified that the number of errors per subject showed no Gaussian distribution. With the Mann-Whitney test, we verified that there was no statistical difference between those who declared they had at least 10/10 visual acuity and those with less than 10/10 (*p*-values 0.227). The *p*-value for the reading errors was 0.227 and for the missed reading errors it was 0.287 *p*-value. The errors were therefore due only to problems of perception during the test. Total errors committed had a median equal to 2 and an interquartile interval equal to 4.75.

Finally, although the focus time of the visor is less than the eye’s accommodation time, the user is watching a scene on a head-mounted display and the time it takes the eye to distinguish two images [31] is less than the focus time of the visor. For this reason, the focusing event could not be fully transparent to the user. This is an interesting perception event that will be further analyzed in future works.

## 4. Discussion

The results of our initial evaluation of the autofocus system show that the proposed system design is useful and almost transparent to the user as it adjusts the focus in 130 ms. Our feasibility study was limited to the magnifying power of the system, but with different optics and sensors, a robust wearable magnification system is clearly possible. The magnification provided by the visor is insufficient for use in microsurgery operations, requiring at least 3× magnification. Poor magnification is due to the focal length of the lens and sensor dimension. The required magnification could be easily achieved by modifying these dimensions. It should also be taken into account that a much-enlarged view significantly reduces the field of view, which can result in the user losing orientation with respect to the working point. To overcome this problem, we evaluated the possibility of the viewer working in OST mode when users need to orient themselves towards the area of interest and in VST mode when the user is already correctly oriented. Magnification over 5× is not of interest as it would not suit a wearable system due to head motion which creates other perceptual issues in handling the magnified image. However, in the context of surgery, an HMD would be advantageous in order to switch between a natural view and a magnified view, and thus, the proposed system would provide the first step towards such a device.

HMDs can also offer Augmented Reality (AR), which is the fusion of real world content and virtual information (e.g., Microsoft Hololens, Microsoft Corporation, Redmond, WA, USA) [7]. HMDs with AR facilities are becoming part of the standard equipment in various fields such as industry, military, automotive, sports and entertainment [32,33]. The technology could also become part of the surgical workflow, where the possible advantages can disrupt surgical outcomes in various surgical specialties since it complements and integrates the concepts of standard surgical navigation based on virtual reality. In the literature, AR HMDs have been used to visualize the 3D anatomy of the patient overlaid onto the surgical field, in order to guide the alignment of bony structures, and in some case to actually guide the surgical gestures [34,35,36].

Pairing the AR with a magnified view would also be interestig as for example presented in [37]. The authors propose combining a Magic Leap One with a surgical loupe, thereby providing both optical magnification and AR features at the same time.

In fact, in the first phase of our study, we also aimed to enrich the system with AR facilities; however, this was hindered by the fact that optics with liquid lenses does not provide the repeatability of the intrinsic parameters of the camera. To check this, we performed several camera calibrations at many distances, with a MATLAB Camera Calibration. One limitation of the current system is thus that, at the moment, AR applications are limited to applications that require low accuracy in registering virtual objects with the real scene. One of our future goals is to use a real-time camera self-calibration procedure to make our VST visor with liquid lenses that are suitable for AR applications and require high precision in the registration process, thus proving both magnified vision and AR in a single device with dynamic focus.

## 5. Conclusions

This work demonstrates that the depth of field in Video See-Through wearable visors can be extended by using optics with liquid lenses along with an open loop control system based on a distance sensor.

Our device can focus at various distances, thus extending the depth of field offered by stable optics (see Table 2, first statements), focus rapidly (roughly 130 ms), provide a nearly transparent focus event for the observer (see Table 2 second statements), and focus without causing image fluctuations. The autofocus system works for visors that provide a magnified view, thus overcoming the current limitation due to the small depth of field of wearable magnifying devices.

## Figures and Tables

**Table 1:** Quantitative test results: numerical evaluation of a focus event duration.

	Near to Far		Far to Near	
	Time (ms)	Frame	Time (ms)	Frame
Mean *	130	11	131	11
Median	129	11	128	11
25°–75°	116–140	10–12	117–148.5	10–12.75
Range	93–175	8–15	93–175	8–18

* Discrete average.

**Table 2:** Likert Questionnaire Qualitative Test Results *.

		First Task		Third Task	
#	Statements	Median IQR (25°–75°)	*p*-Value	Median IQR (25°–75°)	*p*-Value
1	The object in the scene is always in focus.	4 (4–5)	0.003	4 (2–4)	0.157
2	There is no latency during the focusing.	4 (4–5)	0.000	4 (4–4)	0.018
3	I do not perceive any distortion of the scene (i.e., double vision).	2 (2–4)	0.637	4 (2–4)	0.059
4	The image resolution and quality of the. HMD device is suitable for surgical practice.	3 (3–3)	1.000	3 (3–3)	0.020
5	The latency between the real image and the image on the display is suitable for surgical practice.	4 (3–4)	0.001	4 (3–4)	0.001
6	If the HMD device’s zoom was greater (i.e., 2.5× or more), I would use it in the surgical practice (surgeons only).	4 (3.5–4.5)	0.046	4 (3.75–4)	0.025
7	The HMD device provides a stereoscopic view.	4 (4–4)	0.001	4 (4–5)	0.000

* 1 strongly disagree; 2 disagree; 3 neutral; 4 agree; 5 strongly agree.

**Table 3:** Post Hoc Analysis Mann-Whitney Test: Surgeons vs. non-Surgeons *.

		First Task	Third Task
#	Statements	*p*-Value	*p*-Value
1	The object in the scene is always in focus.	0.481	1.000
2	There is no latency during the focusing.	0.386	0.120
3	I do not perceive any distortion of the scene (i.e., double vision).	0.402	0.961
4	The image resolution and quality of the HMD device is suitable for surgical practice.	0.959	0.262
5	The latency between the real image and the image on the display is suitable for surgical practice.	0.106	0.146
6	The HMD device provides a stereoscopic view.	0.811	0.363

* Null hypothesis: due to the medical background of the surgeon group, there is a statistical difference in the perceived value between the surgeon group and the non-surgeon group.

**Table 4:** Post Hoc Analysis Mann-Whitney Test: Experts vs. non-Experts *.

		First Task	Third Task
#	Statements	*p*-Value	*p*-Value
1	The object in the scene is always in focus	0.646	0.467
2	There is no latency during the focusing	0.816	0.394
3	I do not perceived any distortion of the scene (i.e., double vision)	0.719	0.137
4	The image resolution and quality of the HMD device is suitable for surgical practice	0.238	0.576
5	The latency between real image and the image on the display is suitable for surgical practice	0.269	0.373
6	The HMD device provide a stereoscopic view	0.650	0.323

* Null hypothesis: there is a statistical difference in the perceived values between the non-expert and those with a background in the field of HMDs.
